# Crystal structure of bis­(1-methyl-1*H*-imidazole-κ*N*^3^)(5,10,15,20-tetra­phenyl­porphyrinato-κ^4^*N*)iron(II) toluene tris­olvate

**DOI:** 10.1107/S2056989025007121

**Published:** 2025-08-15

**Authors:** Wei Ding, Mingrui He, Jianfeng Li

**Affiliations:** a Beijing Spacecrafts Co., Ltd., Beijing 100094, People’s Republic of China; b State Key Laboratory of Biopharmaceutical Preparation and Delivery Institute of Process Engineering, Chinese Academy of Sciences Beijing 100190 People’s Republic of China; c College of Materials Science and Opto-electronic Technology University of Chinese Academy of Sciences, Huairou Beijing 101408 People’s Republic of China; Texas A & M University, USA

**Keywords:** crystal structure, C—H⋯π inter­action, 1-methyl­imidazole, iron

## Abstract

The title complex, [Fe(C_4_H_6_N_2_)_2_(C_44_H_28_N_4_)]·3C_7_H_8_, possesses inversion symmetry with the iron(II) atom located on a center of symmetry. The metal atom is coordinated in a symmetric octa­hedral geometry by four pyrrole N atoms of the porphyrin ligand in the equatorial plane and two N atoms of 1-methyl­imidazole ligands in the axial sites; the complex crystallizes with three toluene solvent mol­ecules. The average Fe—N_P_ (N_P_ is a porphyrin N atom) bond length is 1.994 (3) Å and the axial Fe—N_Im_ (N_Im_ is an imidazole N atom) bond length is 2.0000 (14) Å. The two 1-methyl­imidazole ligands are mutually parallel. The dihedral angle between the 1-methyl­imidazole plane and the plane of the closest Fe—N_P_ vector is 25.54 (10)°. In the crystal, the only significant inter­molecular inter­actions present are C—H⋯π inter­actions.

## Abstract 2

The title complex, [Fe(C_4_H_6_N_2_)_2_(C_44_H_28_N_4_)]·3C_7_H_8_, possesses inversion symmetry with the iron(II) atom located on a center of symmetry. The metal atom is coordinated in a symmetric octa­hedral geometry by four pyrrole N atoms of the porphyrin ligand in the equatorial plane and two N atoms of 1-methyl­imidazole ligands in the axial sites; the complex crystallizes with three toluene solvent mol­ecules.

## 1. Chemical context

Cytochrome *c* oxidases (C*c*O), a superfamily of proteins, are particularly important in catalyzing O_2_ into water (Ferguson-Miller & Babcock, 1996 ▸; Michel *et al.*, 1998 ▸; Babcock & Wikstrom, 1992 ▸). The best-conserved subunit (subunit I) in C*c*O contains two heme centers (Michel *et al.*, 1998 ▸). The first heme, which is low-spin and bis-histidine coordinated, acts as an electron-input device to the second (Pitcher & Watmough, 2004 ▸). The second heme (heme *a_3_*), which is binuclear with a Cu (Cu_B_) as the other metal, is the site of oxygen reduction. Porphyrin models for both catalytic heme centers have been developed and investigated (Walker, 2004 ▸; Collman *et al.*, 2003 ▸, 2004 ▸; Nakamura, 2006 ▸; Ide *et al.*, 2017 ▸; Ikeue *et al.*, 2011 ▸; Kim *et al.*, 2004 ▸). For the bis-histidine coordinated heme, both ferrous and ferric [Fe^II,III^(Porph)(*L*)_2_]^0,+^ (*L*: planar N-donor ligand) complexes have been studied to understand the correlation between the crystal structures and the spectroscopic properties. Compared to the extensively studied ferric [Fe^III^(Porph)(*L*)_2_]**^+^**, reports on ferrous [Fe^II^(Porph)(*L*)_2_]^0^ complexes are less common. For *d*^6^ Fe^II^ porphyrin species, it has been presumed that the axial ligands would align themselves perpendicularly to maximize the π-bonding between the π* orbitals of the ligands and the filled *d*π orbitals of Fe^II^ (Li *et al.*, 2008 ▸). The first structurally characterized iron(II) bis-imidazole porphyrinate, [Fe(TPP)(1-MeIm)_2_], which was personally communicated (Steffen *et al.*, 1978 ▸), however, showed parallel imidazole orientation with a required symmetry of an inversion center at the iron atom and thus a near planar porphyrin plane (Hu, Roth *et al.*, 2005 ▸). An iron(II) porphyrin complex with mutually perpendicular ligand orientation was eventually achieved in 2005 by using the hindered axial ligands, *i.e.* [Fe(TMP)(2-MeHIm)_2_] (Hu, Noll, *et al.*, 2005 ▸). The crystal structure showed a very ruffled porphyrin core, and the Mössbauer spectra showed a large Δ*E*_Q_ of ∼1.7 mm s^−1^ (Hu, Noll, *et al.*, 2005 ▸). These geometric and Mössbauer properties are in sharp contrast to those of [Fe(Porph)(1-MeIm)_2_] analogues, which showed parallel ligand orientations, near planar porphyrin plane, and Δ*E*_Q_ of ∼1.1 mm s^−1^.
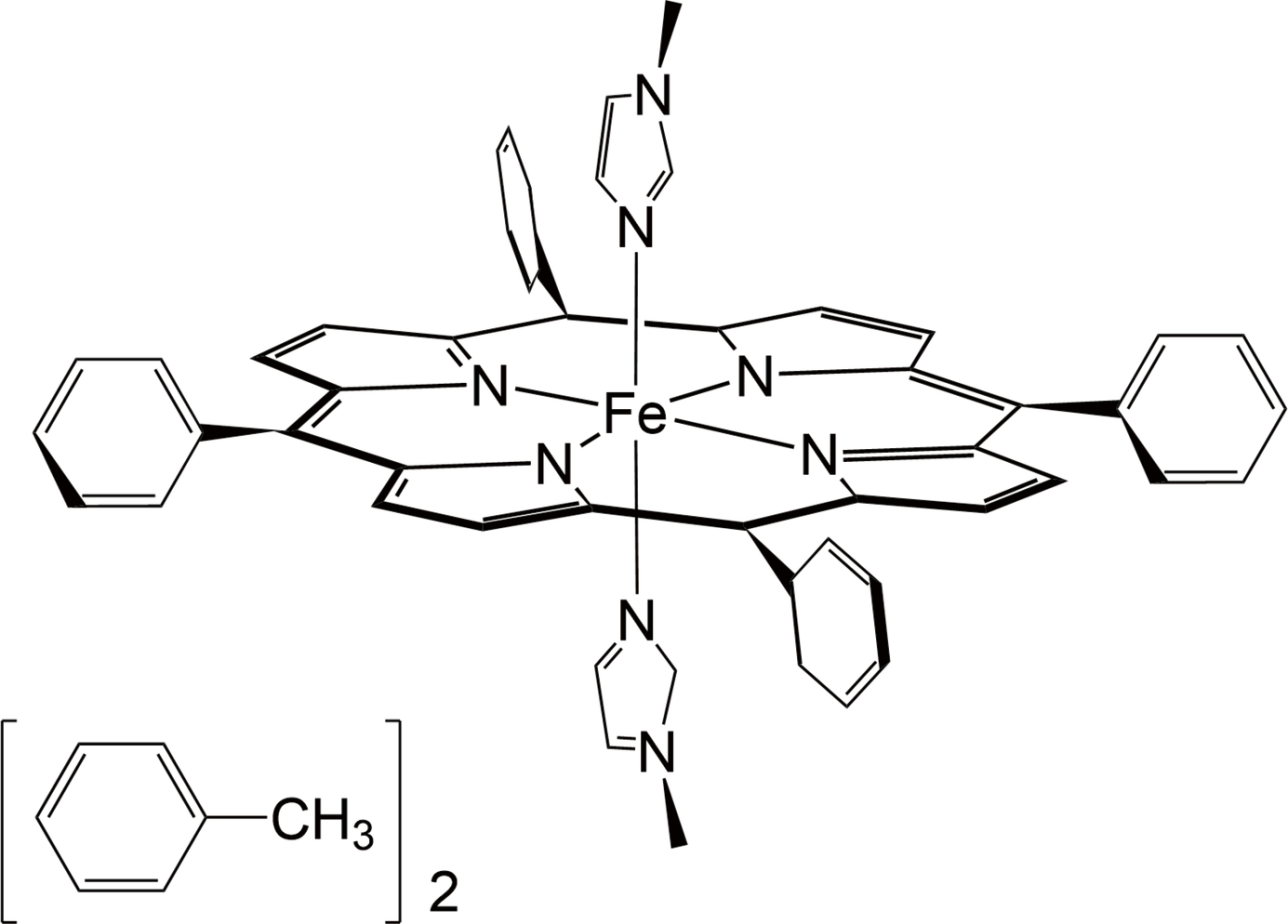


Herein, we report the structural properties of the iron(II) porphyrin complex [Fe^II^(TPP)(1-MeIm)_2_]·3(C_7_H_8_) in which the metal center is octa­hedrally coordinated. Apart from the informally reported [Fe(TPP)(1-MeIm)_2_] (Steffen *et al.*, 1978 ▸), a similar [(Fe(TPP)(1-MeIm)_2_]·2(1-MeIm) where all the 1-MeIm mol­ecules (un)bound to iron were disordered has been reported (Guan *et al.*, 2015 ▸)

## 2. Structural commentary

**Figure 1:**
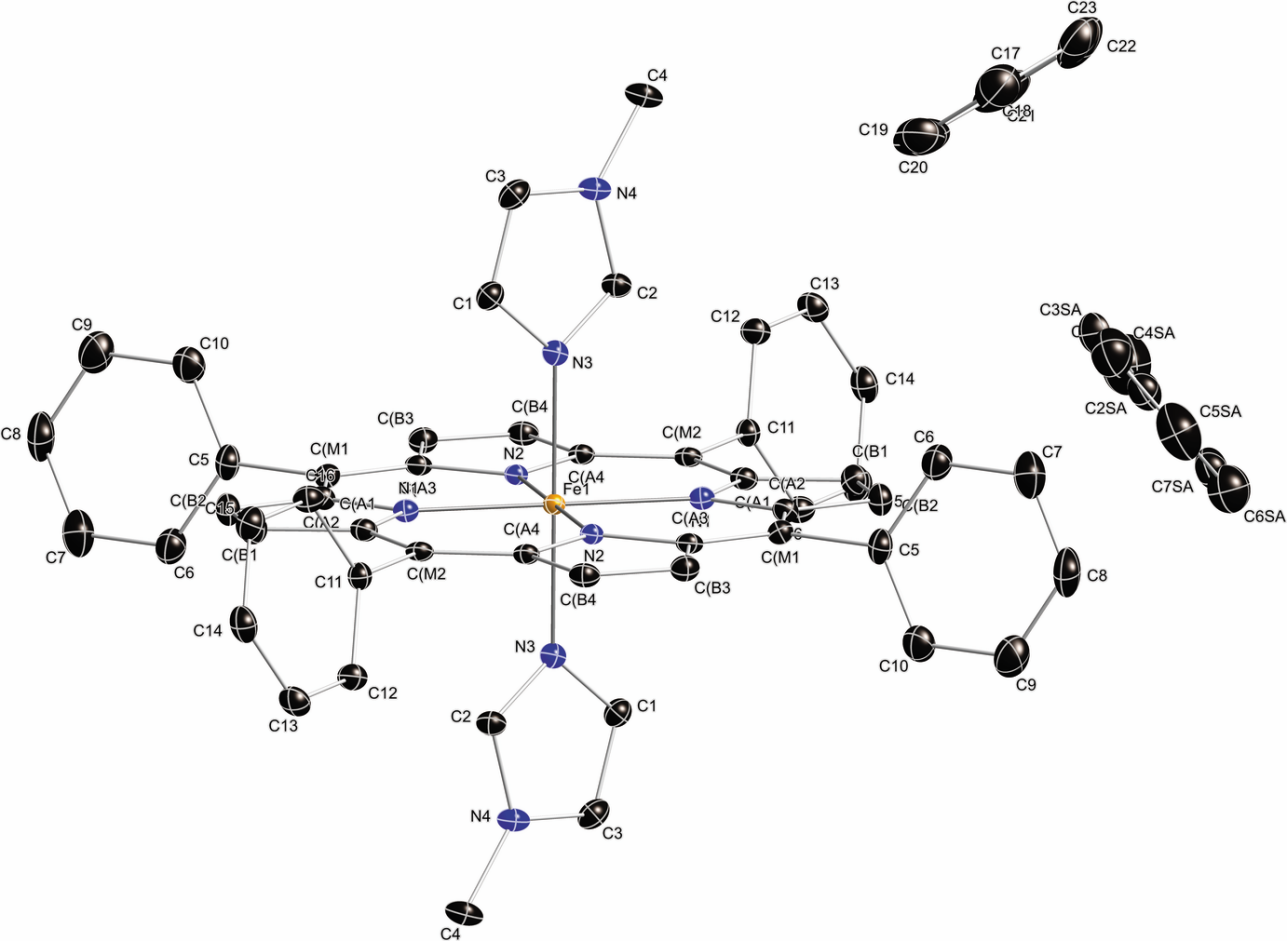
The mol­ecular structure of the title compound, with displacement ellipsoids drawn at the 50% probability level.

**Figure 2:**
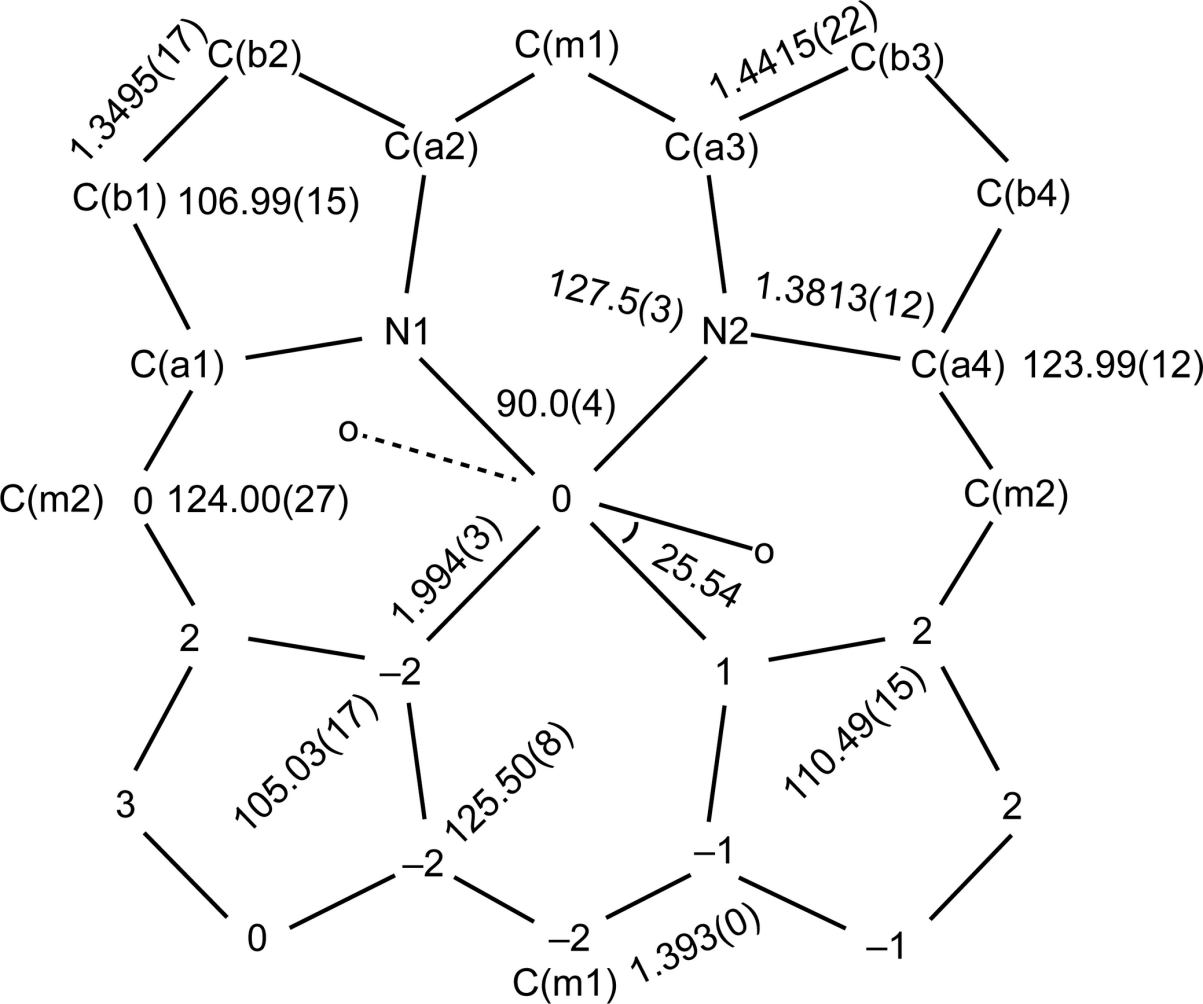
Formal diagram of the porphyrinate core of [Fe^II^(TPP)(1-MeIm)_2_]. Averaged values of the chemically unique bond distances (in Å) and angles (in degrees) are shown. The numbers in parentheses are the esds calculated on the assumption that the averaged values were all drawn from the same population. The perpendicular displacements (in units of 0.01Å) of the porphyrin core atoms from the 24-atom mean plane are also displayed. Positive values of the displacement are towards the hindered porphyrin side, the solid line and dashed line indicate the plane of imidazole on the unhindered porphyrin side.

The asymmetric unit of the title compound (Fig. 1 ▸) contains half of an Fe^II^ porphyrin complex with the iron(II) atom located on an inversion center, one axial 1-methyl­imidazole ligand, as well as one full and one-half toluene solvent mol­ecules. The second toluene was refined at ∼44% and was fixed at 50% occupancy in the final refinement. The two 1-methyl­imidazole ligands of the [Fe^II^(TPP)(1-MeIm)_2_] are mutually parallel, as required by crystallographic symmetry. Additional qu­anti­tative information about the structure is displayed in Fig. 2 ▸, which includes the displacement of each porphyrin core atom (in units of 0.01 Å) from the 24-atom mean plane. The orientation of the 1-methyl­imidazole ligand, including the value of the dihedral angles, is also given. As can be seen in Fig. 2 ▸, the porphyrin core of [Fe(II)(TPP)(1-MeIm)_2_] is near-planar, and the iron(II) atom sits in the 24-atom plane. The displacement of every porphyrin core atom is ≤ 0.03 Å. The average Fe—N_P_ bond length of 1.994 (3) Å is similar to 1.993 (6) Å for [Fe^II^(TpivPP)(1-EtIm)_2_] (Li *et al.*, 2008 ▸) and 1.994 (10) Å for [Fe^II^(TFPPBr_2_)(1-EtIm)_2_] (Hu *et al.*, 2016 ▸), which are typical values for six-coordinate low-spin (porph­inato)iron(II) derivatives (Scheidt *et al.*, 1981 ▸). The axial Fe—N_Im_ bond length is 2.0000 (14) Å, comparable to 1.9970 (12) Å in [(Fe(TPP)(1-MeIm)_2_]·2(1-MeIm) (Guan *et al.*, 2015 ▸). The average N_P_—Fe—N_P_ angle is ideal at 90.0 (4)°. The dihedral angle between the 1-methyl­imidazole plane and the plane of the closest Fe—N_P_ vector is 25.54 (10)°.

## 3. Supra­molecular features

**Figure 3:**
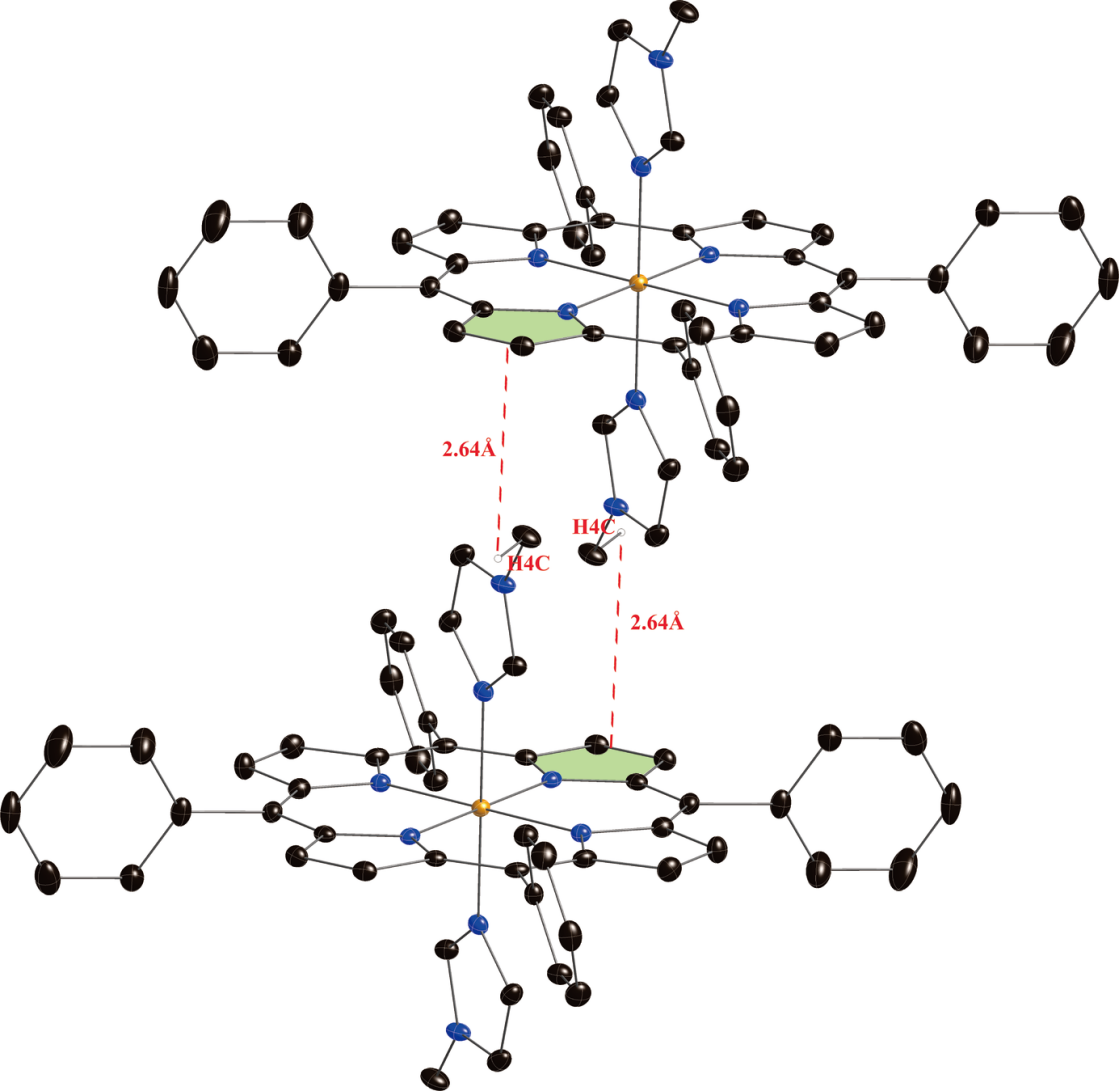
The C—H⋯π inter­actions in the title compound. Dashed lines show the distances between hydrogen atoms of 1-methyl­imidazole and the pyrrole core planes. Solvent (toluene) mol­ecules and other hydrogen atoms have been omitted for clarity.

**Figure 4:**
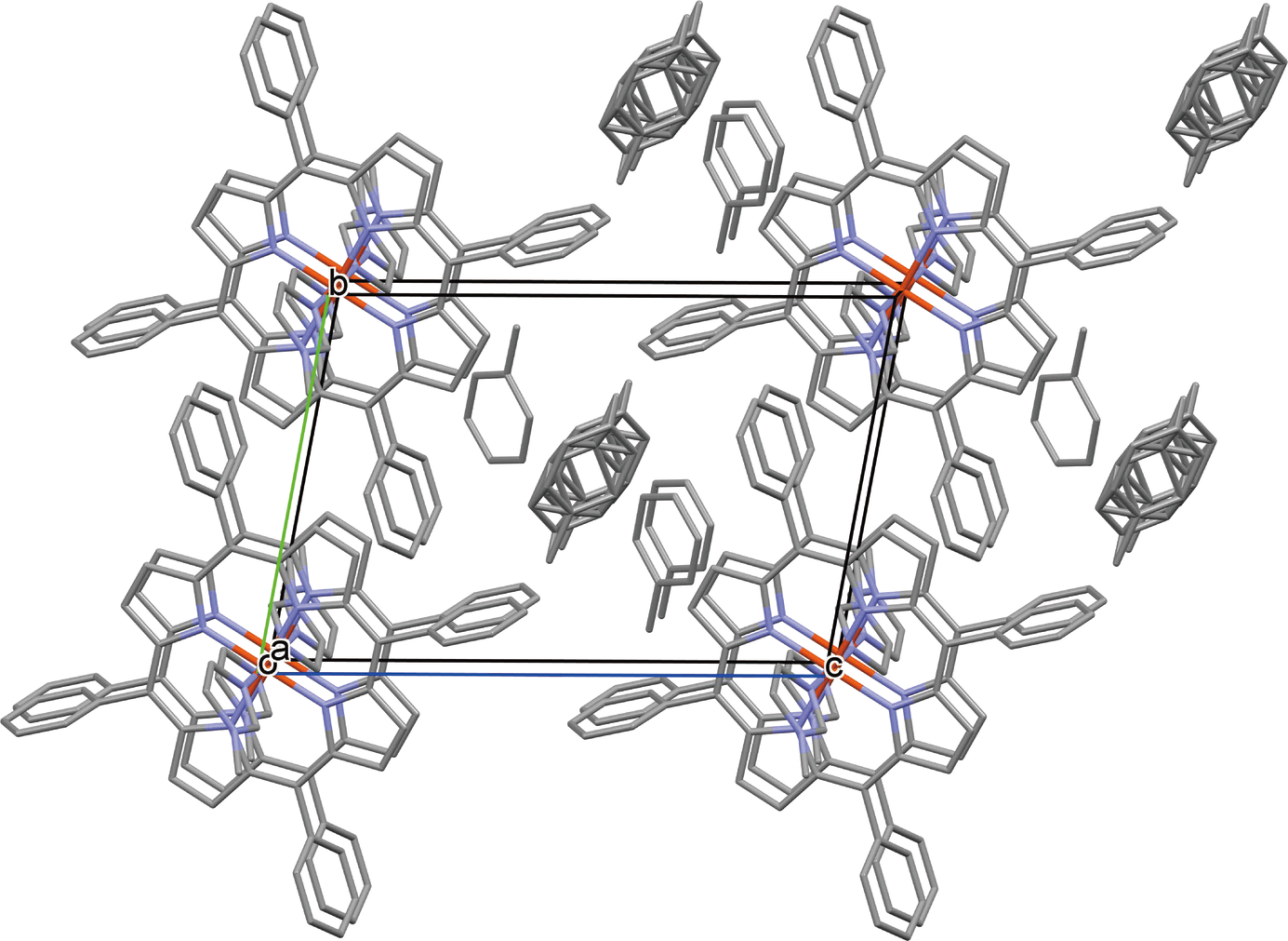
A view of the mol­ecular packing of the title compound in the crystal structure. Hydrogen atoms have been omitted for clarity.

In the title compound, as shown in Fig. 3 ▸, the distance between the hydrogen atom H4*C* (C4) of the methyl group of 1-MeIm and the pyrrole plane of the neighboring porphyrin [the N2, C(A3, C(B3, C(B4, C(A4 ring] is 2.64 (4) Å, smaller than 2.9 Å, which is a limit suggested for the existence of a C—H⋯π inter­action inter­action (Takahashi *et al.*, 2001 ▸). Details of this inter­action are given in Table 1 ▸. The mol­ecular packing is shown in Fig. 4 ▸.

## 4. Synthesis and crystallization

### 4.1. General information

All reactions were carried out using standard Schlenk techniques under argon unless otherwise noted. Toluene and benzene were distilled over sodium, hexa­nes over potassium–sodium alloy and di­chloro­methane (CH_2_Cl_2_) over calcium hydride.

### 4.2. Synthesis of bis­(1-methyl-1*H*-imidazole-κ*N*^3^)(5,10,15,20-tetra­phenyl­porphyrinato-κ^4^*N*)iron(II) toluene trisolv­ate

The purple powder [Fe(TPP)]_2_O (15.9 mg, 0.0234 mmol) was dried in a vacuum for 1h in a Schlenk tube. Benzene (∼5 mL) was transferred into the Schlenk tube by cannula and ethane­thiol (∼2 mL) was added *via* syringe. The mixture was stirred under argon at ambient temperature. After 36 h, the reduction was completed and the solvent was evaporated by pump. Toluene (∼5 mL) was transferred into a Schlenk tube *via* cannula, and 1-MeIm (∼0.5 mL) was added *via* syringe. Hexanes were then allowed to diffuse slowly into the reaction solution. Several weeks later, the block-shaped crystalline product was collected.

## 5. Refinement

Crystal data, data collection and structure refinement details are summarized in Table 2 ▸. H atoms were positioned geometrically (0.95 Å) and refined as riding with *U*_iso_(H) = 1.2*U*_eq_(C).

## Supplementary Material

Crystal structure: contains datablock(s) I. DOI: 10.1107/S2056989025007121/jy2063sup1.cif

Structure factors: contains datablock(s) I. DOI: 10.1107/S2056989025007121/jy2063Isup3.hkl

CCDC reference: 2478878

Additional supporting information:  crystallographic information; 3D view; checkCIF report

Additional supporting information:  crystallographic information; 3D view; checkCIF report

## Figures and Tables

**Table 1:** C—H⋯π inter­action geometry (Å, °) *Cg* is the centroid of the N2, C(A3, C(B3, C(B4, C(A4 ring.

*D*—H⋯*A*	*D*—H	H⋯*A*	*D*⋯*A*	*D*—H⋯*A*
C4—H4*C*⋯*Cg*^i^	0.95 (2)	2.64 (3)	3.408 (2)	137.7 (17)

Symmetry code: (i) 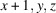.

**Table 2:** Experimental details

Crystal data	
Chemical formula	[Fe(C_4_H_6_N_2_)_2_(C_44_H_28_N_4_)]·3C_7_H_8_
*M*_r_	1109.17
Crystal system, space group	Triclinic, *P* 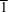
Temperature (K)	100
*a*, *b*, *c* (Å)	8.9075 (4), 10.8001 (5), 15.7095 (8)
α, β, γ (°)	78.759 (2), 81.631 (1), 76.356 (1)
*V* (Å^3^)	1432.58 (12)
*Z*	1
Radiation type	Mo *K*α
μ (mm^−1^)	0.32
Crystal size (mm)	0.30 × 0.19 × 0.05

Data collection	
Diffractometer	Brucker D8 QUEST System
Absorption correction	Multi-scan (*SADABS*; Krause *et al.*, 2015 ▸)
*T*_min_, *T*_max_	0.930, 0.984
No. of measured, independent and observed [*I* > 2σ(*I*)] reflections	30552, 5860, 4702
*R*_int_	0.063
(sin θ/λ)_max_ (Å^−1^)	0.625

Refinement	
*R*[*F*^2^ > 2σ(*F*^2^)], *wR*(*F*^2^), *S*	0.040, 0.095, 1.05
No. of reflections	5860
No. of parameters	403
H-atom treatment	H atoms treated by a mixture of independent and constrained refinement
Δρ_max_, Δρ_min_ (e Å^−3^)	0.35, −0.52

Computer programs: *APEX2*, *SAINT*, *XPREP*, *XP* and *XCIF* (Bruker, 2013 ▸), *SHELXT2014/6* (Sheldrick, 2015*a* ▸), *SHELXL2014/6* (Sheldrick, 2015*b* ▸) and *enCIFer* (Allen *et al.*, 2004 ▸).

## supplementary crystallographic information

### Bis(1-methyl-1H-imidazole-κN3)(5,10,15,20-tetraphenylporphyrinato-κ4N)iron(II) toluene trisolvate Crystal data

**Table:**

[Fe(C_4_H_6_N_2_)_2_(C_44_H_28_N_4_)]·3C_7_H_8_	*Z* = 1
*M**_r_* = 1109.17	*F*(000) = 584
Triclinic, *P*1	*D*_x_ = 1.286 Mg m^−^^3^
*a* = 8.9075 (4) Å	Mo *K*α radiation, λ = 0.71073 Å
*b* = 10.8001 (5) Å	Cell parameters from 9832 reflections
*c* = 15.7095 (8) Å	θ = 2.2–26.4°
α = 78.759 (2)°	µ = 0.32 mm^−^^1^
β = 81.631 (1)°	*T* = 100 K
γ = 76.356 (1)°	Block, purple
*V* = 1432.58 (12) Å^3^	0.30 × 0.19 × 0.05 mm

### Bis(1-methyl-1H-imidazole-κN3)(5,10,15,20-tetraphenylporphyrinato-κ4N)iron(II) toluene trisolvate Data collection

**Table:**

Brucker D8 QUEST System diffractometer	4702 reflections with *I* > 2σ(*I*)
Radiation source: fine-focus sealed tube	*R*_int_ = 0.063
φ and ω scans	θ_max_ = 26.4°, θ_min_ = 2.2°
Absorption correction: multi-scan (SADABS; Krause *et al.*, 2015)	*h* = −11→11
*T*_min_ = 0.930, *T*_max_ = 0.984	*k* = −13→13
30552 measured reflections	*l* = −19→19
5860 independent reflections	

### Bis(1-methyl-1H-imidazole-κN3)(5,10,15,20-tetraphenylporphyrinato-κ4N)iron(II) toluene trisolvate Refinement

**Table:**

Refinement on *F*^2^	Primary atom site location: structure-invariant direct methods
Least-squares matrix: full	Hydrogen site location: mixed
*R*[*F*^2^ > 2σ(*F*^2^)] = 0.040	H atoms treated by a mixture of independent and constrained refinement
*wR*(*F*^2^) = 0.095	*w* = 1/[σ^2^(*F*_o_^2^) + (0.0323*P*)^2^ + 1.1455*P*] where *P* = (*F*_o_^2^ + 2*F*_c_^2^)/3
*S* = 1.05	(Δ/σ)_max_ < 0.001
5860 reflections	Δρ_max_ = 0.35 e Å^−^^3^
403 parameters	Δρ_min_ = −0.52 e Å^−^^3^
0 restraints	

### Bis(1-methyl-1H-imidazole-κN3)(5,10,15,20-tetraphenylporphyrinato-κ4N)iron(II) toluene trisolvate Special details

**Table:**

Geometry. All esds (except the esd in the dihedral angle between two l.s. planes) are estimated using the full covariance matrix. The cell esds are taken into account individually in the estimation of esds in distances, angles and torsion angles; correlations between esds in cell parameters are only used when they are defined by crystal symmetry. An approximate (isotropic) treatment of cell esds is used for estimating esds involving l.s. planes.
Refinement. Refinement of F^2^ against ALL reflections. The weighted R-factor wR and goodness of fit S are based on F^2^, conventional R-factors R are based on F, with F set to zero for negative F^2^. The threshold expression of F^2^ > 2sigma(F^2^) is used only for calculating R-factors(gt) etc. and is not relevant to the choice of reflections for refinement. R-factors based on F^2^ are statistically about twice as large as those based on F, and R- factors based on ALL data will be even larger.

### Bis(1-methyl-1H-imidazole-κN3)(5,10,15,20-tetraphenylporphyrinato-κ4N)iron(II) toluene trisolvate Fractional atomic coordinates and isotropic or equivalent isotropic displacement parameters (Å^2^)

**Table:**

	*x*	*y*	*z*	*U*_iso_*/*U*_eq_	Occ. (<1)
Fe1	0.500000	0.000000	0.000000	0.00883 (10)	
N1	0.47642 (16)	−0.09968 (14)	0.12018 (10)	0.0105 (3)	
N2	0.37571 (16)	0.16048 (14)	0.04180 (9)	0.0098 (3)	
N3	0.69083 (16)	0.04655 (14)	0.02624 (10)	0.0113 (3)	
N4	0.86999 (17)	0.15783 (14)	0.02447 (10)	0.0132 (3)	
C(A1	0.5336 (2)	−0.22972 (17)	0.14810 (12)	0.0119 (4)	
C(A2	0.3964 (2)	−0.05378 (17)	0.19379 (12)	0.0121 (4)	
C(A3	0.31083 (19)	0.17303 (17)	0.12577 (12)	0.0110 (4)	
C(A4	0.34227 (19)	0.28360 (16)	−0.00590 (11)	0.0099 (4)	
C(B1	0.4892 (2)	−0.26427 (18)	0.24014 (12)	0.0153 (4)	
H(BD	0.514690	−0.347544	0.274557	0.018*	
C(B2	0.4046 (2)	−0.15590 (17)	0.26806 (12)	0.0154 (4)	
H(BC	0.358960	−0.148416	0.325887	0.019*	
C(B3	0.2340 (2)	0.30540 (17)	0.13036 (12)	0.0131 (4)	
H(BB	0.179863	0.338354	0.180716	0.016*	
C(B4	0.2537 (2)	0.37342 (17)	0.04928 (12)	0.0129 (4)	
H(BA	0.216325	0.463512	0.031846	0.015*	
C(M1	0.3196 (2)	0.07365 (17)	0.19732 (12)	0.0124 (4)	
C(M2	0.38241 (19)	0.31732 (16)	−0.09500 (12)	0.0106 (4)	
C1	0.7894 (2)	−0.01666 (17)	0.08864 (12)	0.0138 (4)	
H1A	0.781054	−0.095491	0.126425	0.017*	
C2	0.7435 (2)	0.15140 (17)	−0.01067 (12)	0.0130 (4)	
H2A	0.697834	0.214163	−0.056207	0.016*	
C3	0.9003 (2)	0.05152 (18)	0.08763 (12)	0.0155 (4)	
H3A	0.982582	0.029450	0.123762	0.019*	
C4	0.9546 (2)	0.2618 (2)	0.00219 (15)	0.0186 (4)	
H4A	0.933 (3)	0.306 (2)	−0.0580 (16)	0.028 (6)*	
H4B	0.921 (3)	0.322 (2)	0.0432 (15)	0.028 (6)*	
H4C	1.062 (3)	0.223 (2)	0.0037 (15)	0.030 (6)*	
C5	0.2435 (2)	0.10492 (17)	0.28448 (12)	0.0150 (4)	
C6	0.3167 (3)	0.1595 (2)	0.33512 (14)	0.0291 (5)	
H6A	0.416121	0.177704	0.314372	0.035*	
C7	0.2459 (3)	0.1879 (2)	0.41611 (15)	0.0368 (6)	
H7A	0.297289	0.225417	0.450103	0.044*	
C8	0.1021 (3)	0.1619 (2)	0.44731 (14)	0.0284 (5)	
H8A	0.053948	0.181660	0.502528	0.034*	
C9	0.0285 (2)	0.1071 (2)	0.39775 (14)	0.0257 (5)	
H9A	−0.070619	0.088624	0.418934	0.031*	
C10	0.0989 (2)	0.0788 (2)	0.31692 (13)	0.0209 (4)	
H10A	0.047190	0.040844	0.283323	0.025*	
C11	0.3376 (2)	0.45610 (17)	−0.13678 (11)	0.0115 (4)	
C12	0.2191 (2)	0.49692 (17)	−0.19150 (12)	0.0146 (4)	
H12A	0.163160	0.436903	−0.201010	0.018*	
C13	0.1824 (2)	0.62492 (18)	−0.23217 (12)	0.0169 (4)	
H13A	0.102160	0.651872	−0.269763	0.020*	
C14	0.2623 (2)	0.71339 (18)	−0.21817 (12)	0.0173 (4)	
H14A	0.236944	0.800821	−0.246156	0.021*	
C15	0.3795 (2)	0.67427 (18)	−0.16324 (13)	0.0174 (4)	
H15A	0.434025	0.734932	−0.153191	0.021*	
C16	0.4168 (2)	0.54630 (17)	−0.12304 (12)	0.0153 (4)	
H16A	0.497417	0.519748	−0.085648	0.018*	
C1SB	0.7609 (3)	0.8872 (2)	0.32950 (17)	0.0425 (6)	
H23A	0.673860	0.873752	0.373637	0.064*	
H23B	0.812557	0.949129	0.345084	0.064*	
H23C	0.721977	0.921204	0.272446	0.064*	
C2SB	0.8751 (3)	0.7605 (2)	0.32540 (15)	0.0342 (6)	
C3SB	0.8474 (4)	0.6483 (2)	0.38067 (16)	0.0424 (7)	
H18A	0.754871	0.652034	0.419631	0.051*	
C4SB	0.9527 (4)	0.5323 (3)	0.37932 (17)	0.0490 (8)	
H19A	0.933230	0.456915	0.417786	0.059*	
C5SB	1.0869 (3)	0.5256 (3)	0.32193 (19)	0.0465 (7)	
H20A	1.159488	0.445668	0.321101	0.056*	
C6SB	1.1151 (3)	0.6351 (3)	0.26591 (19)	0.0457 (7)	
H21A	1.206433	0.630454	0.225956	0.055*	
C7SB	1.0101 (3)	0.7512 (2)	0.26824 (18)	0.0404 (6)	
H22A	1.030608	0.826308	0.229888	0.048*	
C1SA	0.6379 (7)	0.2791 (6)	0.4655 (4)	0.0481 (15)	0.5
H1S1	0.716863	0.239817	0.505858	0.072*	0.5
H1S2	0.565505	0.221984	0.469878	0.072*	0.5
H1S3	0.688444	0.292076	0.405691	0.072*	0.5
C2SA	0.5491 (4)	0.4093 (2)	0.4890 (2)	0.0299 (11)	0.5
C3SA	0.4311 (5)	0.4818 (3)	0.4397 (2)	0.0337 (16)	0.5
H3SA	0.408661	0.451358	0.391152	0.040*	0.5
C4SA	0.3458 (4)	0.5989 (3)	0.4614 (3)	0.0414 (14)	0.5
H4SA	0.265082	0.648485	0.427675	0.050*	0.5
C5SA	0.3785 (4)	0.6435 (3)	0.5324 (3)	0.0426 (15)	0.5
H5SA	0.320248	0.723487	0.547244	0.051*	0.5
C6SA	0.4966 (5)	0.5709 (4)	0.5817 (2)	0.0405 (14)	0.5
H6SA	0.518992	0.601363	0.630291	0.049*	0.5
C7SA	0.5819 (4)	0.4538 (3)	0.5601 (2)	0.0309 (15)	0.5
H7SA	0.662573	0.404235	0.593768	0.037*	0.5

### Bis(1-methyl-1H-imidazole-κN3)(5,10,15,20-tetraphenylporphyrinato-κ4N)iron(II) toluene trisolvate Atomic displacement parameters (Å^2^)

**Table:**

	*U*^11^	*U*^22^	*U*^33^	*U*^12^	*U*^13^	*U*^23^
Fe1	0.00937 (18)	0.00819 (18)	0.0094 (2)	−0.00260 (14)	−0.00160 (14)	−0.00121 (14)
N1	0.0104 (7)	0.0096 (7)	0.0118 (8)	−0.0021 (6)	−0.0020 (6)	−0.0020 (6)
N2	0.0096 (7)	0.0105 (7)	0.0102 (8)	−0.0041 (6)	−0.0020 (6)	−0.0007 (6)
N3	0.0106 (7)	0.0105 (7)	0.0128 (8)	−0.0012 (6)	−0.0004 (6)	−0.0034 (6)
N4	0.0105 (7)	0.0119 (8)	0.0189 (8)	−0.0032 (6)	−0.0015 (6)	−0.0056 (6)
C(A1	0.0104 (8)	0.0113 (9)	0.0143 (9)	−0.0036 (7)	−0.0043 (7)	0.0007 (7)
C(A2	0.0128 (9)	0.0119 (9)	0.0124 (9)	−0.0049 (7)	−0.0018 (7)	−0.0010 (7)
C(A3	0.0100 (8)	0.0115 (9)	0.0131 (9)	−0.0044 (7)	−0.0010 (7)	−0.0035 (7)
C(A4	0.0088 (8)	0.0094 (8)	0.0125 (9)	−0.0029 (7)	−0.0035 (7)	−0.0013 (7)
C(B1	0.0180 (9)	0.0130 (9)	0.0142 (10)	−0.0043 (7)	−0.0033 (7)	0.0015 (7)
C(B2	0.0188 (10)	0.0149 (9)	0.0112 (9)	−0.0032 (8)	0.0002 (7)	−0.0006 (7)
C(B3	0.0130 (9)	0.0123 (9)	0.0148 (10)	−0.0026 (7)	−0.0011 (7)	−0.0046 (7)
C(B4	0.0122 (9)	0.0091 (8)	0.0182 (10)	−0.0026 (7)	−0.0034 (7)	−0.0029 (7)
C(M1	0.0129 (9)	0.0141 (9)	0.0116 (9)	−0.0044 (7)	−0.0015 (7)	−0.0034 (7)
C(M2	0.0088 (8)	0.0096 (8)	0.0147 (9)	−0.0033 (7)	−0.0050 (7)	−0.0004 (7)
C1	0.0151 (9)	0.0131 (9)	0.0129 (9)	−0.0014 (7)	−0.0040 (7)	−0.0012 (7)
C2	0.0118 (9)	0.0110 (9)	0.0167 (10)	−0.0026 (7)	−0.0026 (7)	−0.0022 (7)
C3	0.0138 (9)	0.0169 (9)	0.0161 (10)	0.0004 (7)	−0.0060 (7)	−0.0050 (8)
C4	0.0137 (10)	0.0177 (10)	0.0268 (12)	−0.0071 (8)	−0.0016 (8)	−0.0058 (9)
C5	0.0222 (10)	0.0092 (9)	0.0106 (9)	0.0001 (7)	−0.0012 (7)	0.0004 (7)
C6	0.0392 (13)	0.0352 (13)	0.0193 (11)	−0.0213 (11)	0.0050 (10)	−0.0095 (10)
C7	0.0580 (16)	0.0420 (14)	0.0202 (12)	−0.0271 (12)	0.0030 (11)	−0.0138 (11)
C8	0.0450 (14)	0.0231 (11)	0.0136 (11)	−0.0051 (10)	0.0062 (9)	−0.0045 (9)
C9	0.0230 (11)	0.0274 (11)	0.0195 (11)	0.0019 (9)	0.0033 (8)	0.0003 (9)
C10	0.0195 (10)	0.0260 (11)	0.0160 (10)	−0.0025 (8)	−0.0016 (8)	−0.0040 (8)
C11	0.0126 (9)	0.0107 (9)	0.0104 (9)	−0.0016 (7)	0.0001 (7)	−0.0019 (7)
C12	0.0140 (9)	0.0133 (9)	0.0174 (10)	−0.0031 (7)	−0.0031 (7)	−0.0035 (7)
C13	0.0187 (10)	0.0147 (9)	0.0152 (10)	−0.0001 (8)	−0.0045 (8)	0.0002 (8)
C14	0.0235 (10)	0.0097 (9)	0.0153 (10)	−0.0016 (8)	0.0019 (8)	0.0004 (7)
C15	0.0221 (10)	0.0124 (9)	0.0193 (10)	−0.0084 (8)	0.0004 (8)	−0.0029 (8)
C16	0.0156 (9)	0.0149 (9)	0.0164 (10)	−0.0049 (7)	−0.0029 (7)	−0.0022 (8)
C1SB	0.0585 (17)	0.0388 (15)	0.0346 (15)	−0.0166 (13)	−0.0153 (12)	−0.0015 (11)
C2SB	0.0528 (15)	0.0299 (12)	0.0282 (13)	−0.0181 (11)	−0.0211 (11)	−0.0011 (10)
C3SB	0.0758 (19)	0.0377 (14)	0.0229 (13)	−0.0234 (14)	−0.0135 (12)	−0.0067 (11)
C4SB	0.096 (2)	0.0317 (14)	0.0292 (14)	−0.0231 (15)	−0.0279 (15)	−0.0003 (11)
C5SB	0.0588 (18)	0.0398 (15)	0.0492 (17)	−0.0079 (13)	−0.0344 (15)	−0.0096 (13)
C6SB	0.0399 (15)	0.0502 (17)	0.0541 (18)	−0.0137 (13)	−0.0221 (13)	−0.0081 (14)
C7SB	0.0460 (15)	0.0368 (14)	0.0443 (16)	−0.0222 (12)	−0.0188 (12)	0.0056 (12)
C1SA	0.048 (3)	0.049 (4)	0.041 (3)	0.000 (3)	0.009 (3)	−0.016 (3)
C2SA	0.028 (3)	0.038 (3)	0.023 (3)	−0.015 (2)	0.001 (2)	0.003 (2)
C3SA	0.041 (4)	0.041 (5)	0.021 (3)	−0.023 (4)	−0.006 (2)	0.011 (3)
C4SA	0.040 (3)	0.046 (4)	0.033 (3)	−0.012 (3)	−0.002 (3)	0.008 (3)
C5SA	0.043 (4)	0.031 (3)	0.043 (4)	−0.003 (3)	0.011 (3)	−0.001 (3)
C6SA	0.040 (3)	0.049 (4)	0.033 (3)	−0.014 (3)	0.003 (3)	−0.009 (3)
C7SA	0.029 (3)	0.042 (5)	0.021 (3)	−0.015 (3)	0.002 (2)	0.003 (3)

### Bis(1-methyl-1H-imidazole-κN3)(5,10,15,20-tetraphenylporphyrinato-κ4N)iron(II) toluene trisolvate Geometric parameters (Å, º)

**Table:**

Fe1—N1	1.9915 (15)	C8—H8A	0.9500
Fe1—N1^i^	1.9916 (15)	C9—C10	1.389 (3)
Fe1—N2^i^	1.9969 (14)	C9—H9A	0.9500
Fe1—N2	1.9969 (14)	C10—H10A	0.9500
Fe1—N3^i^	2.0000 (14)	C11—C12	1.394 (2)
Fe1—N3	2.0000 (14)	C11—C16	1.394 (2)
N1—C(A2	1.381 (2)	C12—C13	1.389 (3)
N1—C(A1	1.383 (2)	C12—H12A	0.9500
N2—C(A3	1.380 (2)	C13—C14	1.384 (3)
N2—C(A4	1.381 (2)	C13—H13A	0.9500
N3—C2	1.325 (2)	C14—C15	1.387 (3)
N3—C1	1.380 (2)	C14—H14A	0.9500
N4—C2	1.346 (2)	C15—C16	1.387 (3)
N4—C3	1.365 (2)	C15—H15A	0.9500
N4—C4	1.456 (2)	C16—H16A	0.9500
C(A1—C(M2^i^	1.394 (2)	C1SB—C2SB	1.507 (4)
C(A1—C(B1	1.442 (3)	C1SB—H23A	0.9800
C(A2—C(M1	1.393 (2)	C1SB—H23B	0.9800
C(A2—C(B2	1.438 (3)	C1SB—H23C	0.9800
C(A3—C(M1	1.392 (3)	C2SB—C7SB	1.388 (4)
C(A3—C(B3	1.443 (2)	C2SB—C3SB	1.397 (3)
C(A4—C(M2	1.392 (2)	C3SB—C4SB	1.379 (4)
C(A4—C(B4	1.443 (2)	C3SB—H18A	0.9500
C(B1—C(B2	1.348 (3)	C4SB—C5SB	1.385 (4)
C(B1—H(BD	0.9500	C4SB—H19A	0.9500
C(B2—H(BC	0.9500	C5SB—C6SB	1.380 (4)
C(B3—C(B4	1.351 (3)	C5SB—H20A	0.9500
C(B3—H(BB	0.9500	C6SB—C7SB	1.380 (4)
C(B4—H(BA	0.9500	C6SB—H21A	0.9500
C(M1—C5	1.500 (2)	C7SB—H22A	0.9500
C(M2—C11	1.499 (2)	C1SA—C2SA	1.526 (6)
C1—C3	1.361 (3)	C1SA—H1S1	0.9800
C1—H1A	0.9500	C1SA—H1S2	0.9800
C2—H2A	0.9500	C1SA—H1S3	0.9800
C3—H3A	0.9500	C2SA—C3SA	1.3900
C4—H4A	1.00 (2)	C2SA—C7SA	1.3900
C4—H4B	0.97 (2)	C3SA—C4SA	1.3900
C4—H4C	0.95 (2)	C3SA—H3SA	0.9500
C5—C6	1.386 (3)	C4SA—C5SA	1.3900
C5—C10	1.386 (3)	C4SA—H4SA	0.9500
C6—C7	1.392 (3)	C5SA—C6SA	1.3900
C6—H6A	0.9500	C5SA—H5SA	0.9500
C7—C8	1.376 (3)	C6SA—C7SA	1.3900
C7—H7A	0.9500	C6SA—H6SA	0.9500
C8—C9	1.377 (3)	C7SA—H7SA	0.9500
			
N1—Fe1—N2	90.33 (6)	C7—C8—H8A	120.3
N1^i^—Fe1—N2	89.67 (6)	C9—C8—H8A	120.3
N2^i^—Fe1—N2	180.0	C8—C9—C10	120.2 (2)
N1—Fe1—N3	90.34 (6)	C8—C9—H9A	119.9
N1^i^—Fe1—N3	89.66 (6)	C10—C9—H9A	119.9
N2^i^—Fe1—N3	91.77 (6)	C5—C10—C9	121.03 (19)
N2—Fe1—N3	88.23 (6)	C5—C10—H10A	119.5
N3^i^—Fe1—N3	180.0	C9—C10—H10A	119.5
C(A2—N1—C(A1	104.88 (14)	C12—C11—C16	118.79 (16)
C(A2—N1—Fe1	127.23 (12)	C12—C11—C(M2	120.94 (15)
C(A1—N1—Fe1	127.89 (12)	C16—C11—C(M2	120.25 (16)
C(A3—N2—C(A4	105.18 (14)	C13—C12—C11	120.32 (17)
C(A3—N2—Fe1	127.17 (12)	C13—C12—H12A	119.8
C(A4—N2—Fe1	127.59 (12)	C11—C12—H12A	119.8
C2—N3—C1	105.36 (15)	C14—C13—C12	120.30 (17)
C2—N3—Fe1	126.00 (12)	C14—C13—H13A	119.9
C1—N3—Fe1	128.53 (12)	C12—C13—H13A	119.9
C2—N4—C3	107.28 (15)	C13—C14—C15	119.95 (17)
C2—N4—C4	126.43 (16)	C13—C14—H14A	120.0
C3—N4—C4	126.24 (16)	C15—C14—H14A	120.0
N1—C(A1—C(M2^i^	125.38 (16)	C16—C15—C14	119.77 (17)
N1—C(A1—C(B1	110.47 (15)	C16—C15—H15A	120.1
C(M2^i^—C(A1—C(B1	124.11 (16)	C14—C15—H15A	120.1
N1—C(A2—C(M1	125.52 (16)	C15—C16—C11	120.86 (17)
N1—C(A2—C(B2	110.63 (15)	C15—C16—H16A	119.6
C(M1—C(A2—C(B2	123.85 (17)	C11—C16—H16A	119.6
N2—C(A3—C(M1	125.50 (16)	C2SB—C1SB—H23A	109.5
N2—C(A3—C(B3	110.59 (15)	C2SB—C1SB—H23B	109.5
C(M1—C(A3—C(B3	123.90 (16)	H23A—C1SB—H23B	109.5
N2—C(A4—C(M2	125.60 (16)	C2SB—C1SB—H23C	109.5
N2—C(A4—C(B4	110.28 (15)	H23A—C1SB—H23C	109.5
C(M2—C(A4—C(B4	124.08 (16)	H23B—C1SB—H23C	109.5
C(B2—C(B1—C(A1	106.95 (16)	C7SB—C2SB—C3SB	118.1 (2)
C(B2—C(B1—H(BD	126.5	C7SB—C2SB—C1SB	121.9 (2)
C(A1—C(B1—H(BD	126.5	C3SB—C2SB—C1SB	120.1 (2)
C(B1—C(B2—C(A2	107.07 (16)	C4SB—C3SB—C2SB	120.8 (3)
C(B1—C(B2—H(BC	126.5	C4SB—C3SB—H18A	119.6
C(A2—C(B2—H(BC	126.5	C2SB—C3SB—H18A	119.6
C(B4—C(B3—C(A3	106.78 (16)	C3SB—C4SB—C5SB	120.0 (3)
C(B4—C(B3—H(BB	126.6	C3SB—C4SB—H19A	120.0
C(A3—C(B3—H(BB	126.6	C5SB—C4SB—H19A	120.0
C(B3—C(B4—C(A4	107.16 (16)	C6SB—C5SB—C4SB	120.0 (3)
C(B3—C(B4—H(BA	126.4	C6SB—C5SB—H20A	120.0
C(A4—C(B4—H(BA	126.4	C4SB—C5SB—H20A	120.0
C(A3—C(M1—C(A2	124.24 (17)	C7SB—C6SB—C5SB	119.7 (3)
C(A3—C(M1—C5	118.42 (16)	C7SB—C6SB—H21A	120.2
C(A2—C(M1—C5	117.33 (16)	C5SB—C6SB—H21A	120.2
C(A4—C(M2—C11	118.82 (15)	C6SB—C7SB—C2SB	121.4 (2)
C(A1^i^—C(M2—C11	117.40 (16)	C6SB—C7SB—H22A	119.3
C3—C1—N3	109.40 (16)	C2SB—C7SB—H22A	119.3
C3—C1—H1A	125.3	C2SA—C1SA—H1S1	109.5
N3—C1—H1A	125.3	C2SA—C1SA—H1S2	109.5
N3—C2—N4	111.47 (16)	H1S1—C1SA—H1S2	109.5
N3—C2—H2A	124.3	C2SA—C1SA—H1S3	109.5
N4—C2—H2A	124.3	H1S1—C1SA—H1S3	109.5
C1—C3—N4	106.49 (16)	H1S2—C1SA—H1S3	109.5
C1—C3—H3A	126.8	C3SA—C2SA—C7SA	120.0
N4—C3—H3A	126.8	C3SA—C2SA—C1SA	119.0 (4)
N4—C4—H4A	107.0 (13)	C7SA—C2SA—C1SA	121.0 (4)
N4—C4—H4B	110.5 (13)	C4SA—C3SA—C2SA	120.0
H4A—C4—H4B	110.6 (18)	C4SA—C3SA—H3SA	120.0
N4—C4—H4C	107.0 (14)	C2SA—C3SA—H3SA	120.0
H4A—C4—H4C	110.6 (19)	C3SA—C4SA—C5SA	120.0
H4B—C4—H4C	111.1 (19)	C3SA—C4SA—H4SA	120.0
C6—C5—C10	118.25 (18)	C5SA—C4SA—H4SA	120.0
C6—C5—C(M1	120.81 (17)	C6SA—C5SA—C4SA	120.0
C10—C5—C(M1	120.94 (17)	C6SA—C5SA—H5SA	120.0
C5—C6—C7	120.6 (2)	C4SA—C5SA—H5SA	120.0
C5—C6—H6A	119.7	C5SA—C6SA—C7SA	120.0
C7—C6—H6A	119.7	C5SA—C6SA—H6SA	120.0
C8—C7—C6	120.5 (2)	C7SA—C6SA—H6SA	120.0
C8—C7—H7A	119.7	C6SA—C7SA—C2SA	120.0
C6—C7—H7A	119.7	C6SA—C7SA—H7SA	120.0
C7—C8—C9	119.4 (2)	C2SA—C7SA—H7SA	120.0
			
C(A2—N1—C(A1—C(M2^i^	177.45 (16)	N3—C1—C3—N4	0.2 (2)
Fe1—N1—C(A1—C(M2^i^	−1.5 (2)	C2—N4—C3—C1	0.0 (2)
C(A2—N1—C(A1—C(B1	−0.38 (18)	C4—N4—C3—C1	177.35 (17)
Fe1—N1—C(A1—C(B1	−179.34 (12)	C(A3—C(M1—C5—C6	−80.8 (2)
C(A1—N1—C(A2—C(M1	179.45 (17)	C(A2—C(M1—C5—C6	98.5 (2)
Fe1—N1—C(A2—C(M1	−1.6 (3)	C(A3—C(M1—C5—C10	99.9 (2)
C(A1—N1—C(A2—C(B2	0.27 (19)	C(A2—C(M1—C5—C10	−80.8 (2)
Fe1—N1—C(A2—C(B2	179.25 (11)	C10—C5—C6—C7	−0.4 (3)
C(A4—N2—C(A3—C(M1	−178.01 (16)	C(M1—C5—C6—C7	−179.7 (2)
Fe1—N2—C(A3—C(M1	−0.7 (2)	C5—C6—C7—C8	0.1 (4)
C(A4—N2—C(A3—C(B3	0.82 (18)	C6—C7—C8—C9	0.2 (4)
Fe1—N2—C(A3—C(B3	178.13 (11)	C7—C8—C9—C10	−0.2 (3)
C(A3—N2—C(A4—C(M2	−178.57 (16)	C6—C5—C10—C9	0.4 (3)
Fe1—N2—C(A4—C(M2	4.1 (2)	C(M1—C5—C10—C9	179.71 (18)
C(A3—N2—C(A4—C(B4	−0.68 (18)	C8—C9—C10—C5	−0.1 (3)
Fe1—N2—C(A4—C(B4	−177.98 (11)	C(A4—C(M2—C11—C12	−107.6 (2)
N1—C(A1—C(B1—C(B2	0.3 (2)	C(A1^i^—C(M2—C11—C12	74.1 (2)
C(M2^i^—C(A1—C(B1—C(B2	−177.51 (17)	C(A4—C(M2—C11—C16	73.9 (2)
C(A1—C(B1—C(B2—C(A2	−0.2 (2)	C(A1^i^—C(M2—C11—C16	−104.3 (2)
N1—C(A2—C(B2—C(B1	−0.1 (2)	C16—C11—C12—C13	0.7 (3)
C(M1—C(A2—C(B2—C(B1	−179.26 (17)	C(M2—C11—C12—C13	−177.76 (17)
N2—C(A3—C(B3—C(B4	−0.7 (2)	C11—C12—C13—C14	−0.5 (3)
C(M1—C(A3—C(B3—C(B4	178.19 (16)	C12—C13—C14—C15	−0.1 (3)
C(A3—C(B3—C(B4—C(A4	0.21 (19)	C13—C14—C15—C16	0.5 (3)
N2—C(A4—C(B4—C(B3	0.29 (19)	C14—C15—C16—C11	−0.3 (3)
C(M2—C(A4—C(B4—C(B3	178.22 (16)	C12—C11—C16—C15	−0.3 (3)
N2—C(A3—C(M1—C(A2	0.1 (3)	C(M2—C11—C16—C15	178.17 (17)
C(B3—C(A3—C(M1—C(A2	−178.55 (16)	C7SB—C2SB—C3SB—C4SB	1.1 (3)
N2—C(A3—C(M1—C5	179.32 (16)	C1SB—C2SB—C3SB—C4SB	−178.3 (2)
C(B3—C(A3—C(M1—C5	0.6 (3)	C2SB—C3SB—C4SB—C5SB	−0.9 (4)
N1—C(A2—C(M1—C(A3	1.1 (3)	C3SB—C4SB—C5SB—C6SB	−0.1 (4)
C(B2—C(A2—C(M1—C(A3	−179.87 (17)	C4SB—C5SB—C6SB—C7SB	0.8 (4)
N1—C(A2—C(M1—C5	−178.14 (16)	C5SB—C6SB—C7SB—C2SB	−0.5 (4)
C(B2—C(A2—C(M1—C5	0.9 (3)	C3SB—C2SB—C7SB—C6SB	−0.4 (4)
N2—C(A4—C(M2—C(A1^i^	−1.6 (3)	C1SB—C2SB—C7SB—C6SB	179.0 (2)
C(B4—C(A4—C(M2—C(A1^i^	−179.22 (16)	C7SA—C2SA—C3SA—C4SA	0.0
N2—C(A4—C(M2—C11	−179.75 (15)	C1SA—C2SA—C3SA—C4SA	178.2 (4)
C(B4—C(A4—C(M2—C11	2.6 (2)	C2SA—C3SA—C4SA—C5SA	0.0
C2—N3—C1—C3	−0.3 (2)	C3SA—C4SA—C5SA—C6SA	0.0
Fe1—N3—C1—C3	−176.66 (12)	C4SA—C5SA—C6SA—C7SA	0.0
C1—N3—C2—N4	0.3 (2)	C5SA—C6SA—C7SA—C2SA	0.0
Fe1—N3—C2—N4	176.76 (11)	C3SA—C2SA—C7SA—C6SA	0.0
C3—N4—C2—N3	−0.2 (2)	C1SA—C2SA—C7SA—C6SA	−178.2 (4)
C4—N4—C2—N3	−177.55 (16)		

Symmetry code: (i) −*x*+1, −*y*, −*z*.

### Bis(1-methyl-1H-imidazole-κN3)(5,10,15,20-tetraphenylporphyrinato-κ4N)iron(II) toluene trisolvate Hydrogen-bond geometry (Å, º)

*Cg* is the centroid of the N2, C(A3, C(B3, C(B4, C(A4 ring.

**Table:**

*D*—H···*A*	*D*—H	H···*A*	*D*···*A*	*D*—H···*A*
C4—H4*C*···*Cg*^ii^	0.95 (2)	2.64 (3)	3.408 (2)	137.7 (17)

Symmetry code: (ii) *x*+1, *y*, *z*.
